# Prosthetic Porcelain

**Published:** 1904-10-15

**Authors:** N. S. Jenkins

**Affiliations:** Dresden


### PROSTHETIC PORCELAIN.

BY N. S. JENKINS, D. D. S., DRESDEN.

This new body is designed to be used with artificial teeth and is not intended for inlay purposes. It melts well within the fusing point of gold. This is a matter of great importance. It is comparatively easy to make a dental porcelain which fuses above the melting point of gold, but such a body renders the use of pure gold solder inadvisable, and a body which fuses below, but still very near the gold fusing point, can only be employed with gold solder under hampering precautions. Prosthetic porcelain unites perfectly well with any artificial teeth; indeed, its union is so close that the body of the tooth will break under the hammer before it will lose its new attachment. It is very strong, affording a basis to which a small section of a tooth can be united without danger of fracture and, in cases of a close bite, it permits porcelain restoration where such restoration would formerly have been impossible. It completely obviates the necessity of disfiguring the mouth with the gleam of gold in crowns and bridges and, by its use, the cleanest and most natural work can be made, and that with mathematical accuracy. Short, or deeply-imbedded roots can, if reasonably firm, be utilized to great advantage by its judicious application. In fact, it enables the skillful and patient operator to achieve results vastly superior to any hitherto attainable, for it gives him a material which can be carried into any deep depression, crevice or corner; which can be molded to any desired shape; which gives any desired color; which sustains any desired pressure, and which permits of grinding and subsequent polishing, because of its great density.

You are familiar with continuous gum work and know how difficult it has been, under the old methods, to avoid cracks, blow holes and bubbles, especially at the margins.

If you will examine the specimens with a magnifying glass, you will observe that the prosthetic porcelain melts solidly down to the finest edges, being as free from porosity at the margins of the platinum as in the centre of the piece. This result is always obtainable, because this porcelain has been reduced to manageable proportions. It is only an expert, and, even he, but through unfailing watchfulness and infallible judgment, who can produce such results with a porcelain so undeveloped that it can be melted only at a high temperature; but with this body the general practitioner can safely essay these operations, since there is no step of the process which is not under perfect control.

I pass over the ordinary procedure, except to remark that, in my opinion, the covering of the lingual surface of an upper denture with porcelain is undesirable. The great objection to this beautiful and cleanly work in the upper jaw is its weight. By using platinum, alloyed with 10 per cent irridium, a thin but very stiff plate can be stamped. Of course, it requires care and time, but with sharp dies, an accurate fit is always obtainable. If, then, a still thinner rim is soldered labially and lingually to the plate, leaving space sufficient to solder the teeth in their places and allowing room enough for prosthetic porcelain to flow, a comparatively light denture may be constructed and yet one so strong, that there is no necessity for covering the lingual surface with porcelain.

Four packings and meltings are necessary to fill out and unite the different portions of the skeleton, for the office of this porcelain is chiefly to build out and to support, not primarily to furnish an uncertain and, at best, somewhat imperfect representation of the natural teeth, although it can also be used for this purpose. However great may be his skill and taste, no man can always carve and bake teeth, perfect as to form, size and color. He can always, however, select out of the vast variety furnished by the great manufacturers, teeth which can, in all these respects, be made to conform to the natural organs, and, when these teeth are reinforced by prosthetic porcelain, the results are as practical as they are beautiful. They are also results which may be obtained by all skillful dentists, for this material is designed to make more efficient the aids which commercialism has given us and to make possible, for the ordinary practitioner, operations hitherto performed only by the chosen few. For continuous-gum work there is only required the skeleton of the platinum irridium plate, with the teeth soldered in their places with pure gold, and then the intervening spaces and the failing contours can be filled out, with prosthetic porcelain, with ease and certainty. If the skeleton is anatomically correct and its parts equally strong, the clothing with form and color, by the addition of prosthetic porcelain, is like calling into life the inanimate clay.

The use of this new porcelain in crown and bridge-work will possess for you peculiar attractions; for, even under the old conditions, the restorations you have been accustomed to make, to spare your patients the calamity of wearing plates, have been amongst your greatest triumphs. You will therefore be interested to see how completely all evidence of artificiality can be avoided by the system I have the pleasure to present to you. My crowns and bridges are not only more beautiful, but also far stronger than those made under the old conditions. In those rare cases when labial or buccal bands are unavoidable, you will see how they may be concealed. Where, as is so frequently the case, bands are objectionable, you will observe how they may be dispensed with and yet sufficient strength be secured. In close bites, where otherwise the only chance for a masticating occlusion would be disfiguring and unstable gold or amalgam, you will see restorations perfect in form and color and indestructible in substance, although so thin in bulk. To cut out a film of an artificial bicuspid or molar crown, and set it in a platinum box, itself concealed by the bulging prosthetic porcelain which fills its recesses and holds securely the segment of the crown, either for a single tooth or in a bridge, is an operation as practical as it is beautiful. Here is no wasting through the force of mastication. Here is no shrinkage or roughening of surface or edges. Here is no favorite grazing ground for noxious micro-organisms. Here is no glimmer of gold to proclaim past unsoundness to the inquisitive eye. But here is a process so natural and so logical, so efficient, so full of blessing to the patient and so rich in advantage to the patient's doctor, that we may well pause for a time, until we have assimilated some of its possibilities, before we pass on to the unknown triumphs of the future.—*Sept. Review.*

